# Nemolizumab-induced psoriasiform dermatitis

**DOI:** 10.1016/j.jdcr.2025.05.057

**Published:** 2025-09-10

**Authors:** Gilles Absil, Patrick Collins, Orphal Colleye, Bita Dezfoulian, Arjen F. Nikkels

**Affiliations:** a Department of Dermatology, CHU du Sart Tilman, University of Liège, Liège, Belgium; b Department of Dermatopathology, CHU du Sart Tilman, University of Liège, Liège, Belgium

**Keywords:** atopic dermatitis, cutaneous adverse drug reaction, nemolizumab, psoriasiform dermatitis

## Introduction

Atopic dermatitis (AD) is a common chronic pruritic, relapsing, inflammatory skin disease. The advances in the understanding of AD pathogenesis led to the development of novel targeted therapies. Nemolizumab, a humanized monoclonal antibody targeting the interleukin-31 receptor α, is currently indicated for the treatment of AD as well as for prurigo nodularis. Preliminary data relate significant improvement in both EASI and pruritus scores.^1^^,^^2^ The principal cutaneous adverse drug reaction (CADR) was an exacerbation of AD, reported in 23% of patients in the pivotal trial.^1^

Two patients with severe AD developed, after the administration of nemolizumab, a severe and extensive psoriasiform eruption.

## Case report

### Patient 1

A 49-year-old woman with severe AD since childhood presented with a diffuse non-pruritic eruption that appeared 3 months after the initiation of nemolizumab 30 mg/mo. Prior treatment for AD included topical corticosteroids, phototherapy, cyclosporin, interrupted due to severe headaches and nausea, and dupilumab with severe treatment-related bilateral conjunctivitis and keratoconus.

She denied the intake of any new medication or recent illness or stressful events. No systemic symptoms were evidenced. The eruption appeared progressively 4 weeks prior to the visit. Physical examination revealed diffuse and confluent erythematous and squamous patches involving the trunk and the extremities, different in appearance and localization from her usual AD lesions.

The histopathologic examination of a 4-mm punch biopsy revealed a spongiotic and psoriasiform dermatitis associated with focal parakeratosis, intracorneal pustulosis and a superficial perivascular lymphocytic infiltrate with eosinophils compatible with psoriasiform toxic CADR.

Nemolizumab was interrupted and replaced by upadacitinib leading to a progressive clearance of the CADR and improvement of AD.

### Patient 2

**Fig 1:**
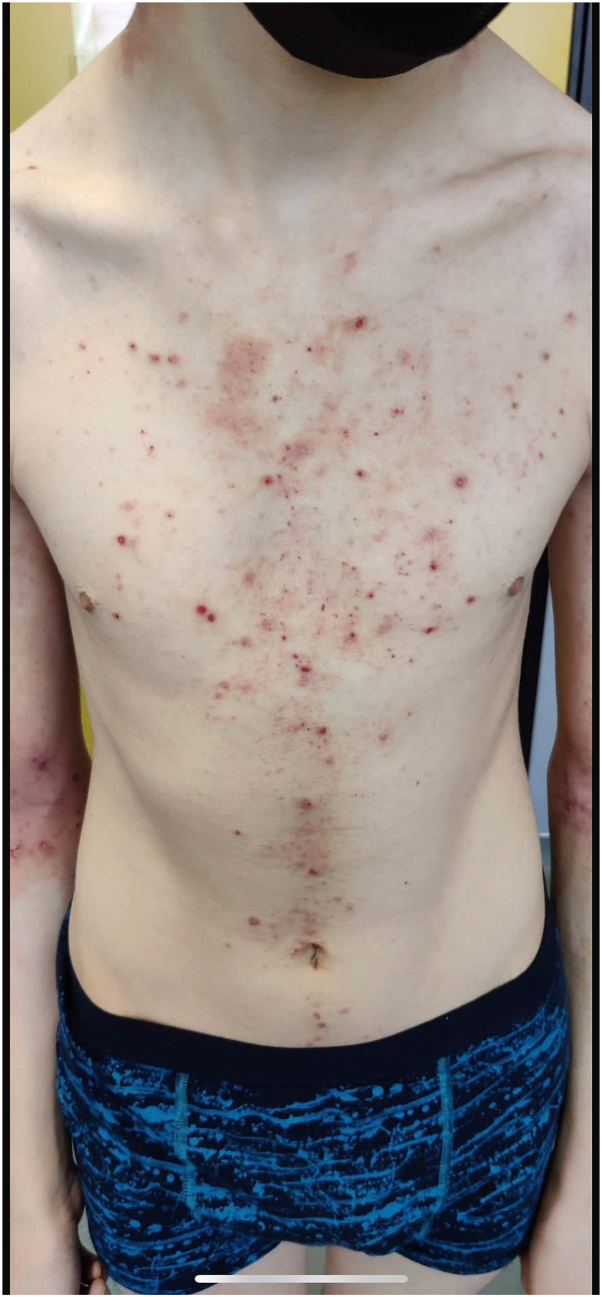
Patient 2 presenting AD prior to nemolizumab.

**Fig 2:**
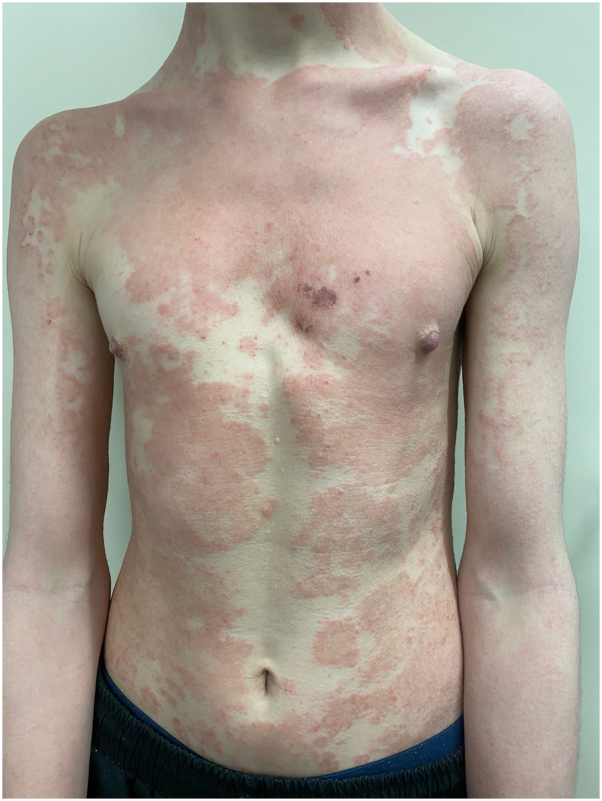
Patient 2. Psoriasiform nemolizumab-associated eruption of the trunk.

A 16-year-old boy suffered from severe AD (Fig 1) since early childhood. Previous AD treatments included topical corticosteroids and narrow-band UVB phototherapy. Due to the severity of his AD, the patient accepted to participate in a nemolizumab AD trial. About 4 months after the introduction of nemolizumab 30 mg once monthly, he presented with an extensive non-pruritic erythematous, slightly squamous dermatitis involving principally the trunk, upper arms and upper limbs that appeared progressively (Fig 2). The eruption was different from his usual AD lesions (Fig 1).

**Fig 3:**
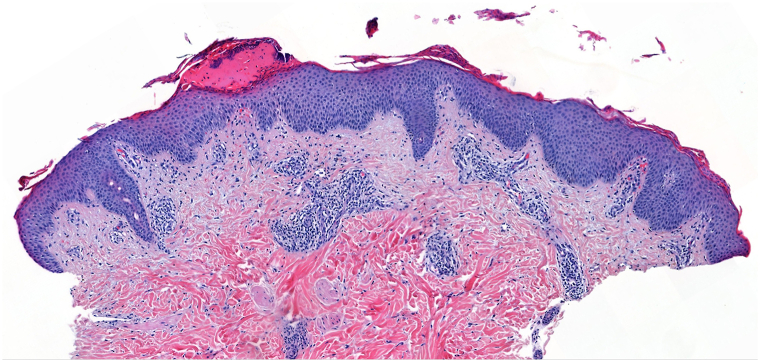
Patient 2. JAK-inhibitor-associated acne with resolution of AD and nemolizumab-associated psoriasiform eruption.

Histopathology evidenced a psoriasiform dermatitis with a superficial perivascular lymphocytic infiltrate with eosinophils compatible with a drug-induced psoriasiform dermatitis (Fig 3).

An intramuscular injection of methylprednisolone was administered due to the severity of the CADR and nemolizumab was interrupted. One week later, the skin lesions had disappeared.

**Fig 4:**
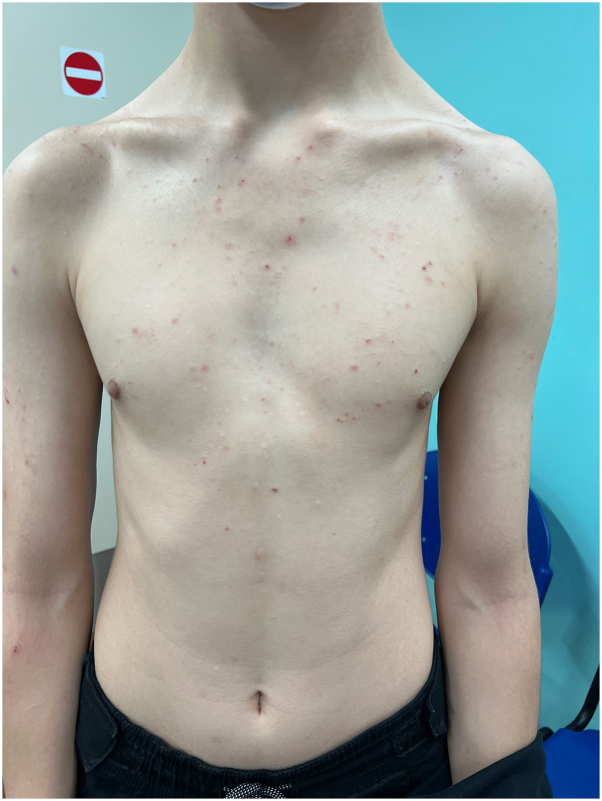
Histology demonstrating a psoriasiform dermatitis (H/E, 10×).

Nemolizumab was replaced by upadacitinib leading to a marked improvement of his AD but he developed JAK-inhibitor-associated acne (Fig 4).

## Discussion

According to the nemolizumab phase III 16-week clinical trial in 143 patients, the most common adverse events were worsening of AD (23%) and injection-related reactions (8%). No other severe eruptions were reported. It is also mentioned that although patients reported a worsening of AD, a significative reduction of pruritus was measured using the visual analog score (VAS). This intriguing phenomenon could not be explained by the authors.^1^

Interleukin-31 (IL-31) is a cytokine of the IL-6 family mainly produced by CD4+ Th2 cells^3^ It binds to the IL-31R complex, which is composed of IL-31 receptor α (IL-31RA) and oncostatin M receptor β (OSMRβ), mediating the induction of pruritus and the release of numerous proinflammatory mediators such as IL-4, IlL-6, IL-8, IL-13, IL-16, and IL-32^4^ High serum levels of IL-31 have been demonstrated in both intrinsic and extrinsic AD, and these levels seem to correlate with disease activity.^5^^,^^6^

Psoriasis is an inflammatory dermatosis with a Th17 cytokine profile. Higher serum levels of IL-31 have also been described in this pathology as well as in psoriatic arthritis compared to controls.^7^, ^8^, ^9^ Thus, it remains unclear why our 2 patients developed this paradoxical psoriasiform eruption. Nevertheless, AD is an entity with a great heterogeneity, both genetically and phenotypically. We postulate that in a specific subset of patients with AD, the IL-31 pathway inhibition triggers a disruption of the cytokine balance upregulating the Th17 profile, responsible for the psoriasiform skin lesions. One prior case with similar features has been reported.^10^

This case demonstrates that severe psoriasiform CADR is a possible new drug adverse effect of IL-31 inhibition therapy. The concerning rate of skin eruptions described as AD worsening without significant pruritus in the nemolizumab clinical trial made us wonder whether at least some of those patients did not have in fact psoriasiform CADR. We suggest that a punch biopsy should be performed in cases of atypical lesions or atypical localization of skin lesions in patients with AD treated with nemolizumab to rule out CADR. Further investigations are necessary to determine which subsets of patients with AD are at risk to develop this eruption.

## Conflicts of interest

None disclosed.
